# Mycoheterotrophic continuum in rhizoctonia associations: genetic divergence and carbon acquisition variation among *Odontochilus* orchids

**DOI:** 10.1093/aob/mcaf100

**Published:** 2025-05-14

**Authors:** Kenji Suetsugu, Hidehito Okada, Shun K Hirota, Yoshihisa Suyama

**Affiliations:** Department of Biology, Graduate School of Science, Kobe University, 1-1 Rokkodai, Nada-ku, Kobe, Hyogo 657-8501, Japan; Institute for Advanced Research, Kobe University, 1-1 Rokkodai, Nada-ku, Kobe, Hyogo 657-8501, Japan; Department of Biology, Graduate School of Science, Kobe University, 1-1 Rokkodai, Nada-ku, Kobe, Hyogo 657-8501, Japan; Botanical Gardens, Osaka Metropolitan University, 2000 Kisaichi, Katano, Osaka 576-0004, Japan; Graduate School of Symbiotic Systems Science and Technology, Fukushima University, Fukushima, Fukushima 960-1296, Japan; Field Science Center, Graduate School of Agricultural Science, Tohoku University, 232-3 Yomogida, Naruko-onsen, Osaki, Miyagi 989-6711, Japan

**Keywords:** Ceratobasidiaceae, coralloid rhizomes, genetic differentiation, MIG-seq, mycoheterotrophy, orchids, phylogenetics, stable isotopes

## Abstract

**Background and Aims:**

Mycoheterotrophy is a nutritional strategy in which plants obtain carbon and essential nutrients from fungal partners. Comparative studies of closely related taxa differing in mycoheterotrophic dependence offer important insights into the evolutionary transitions underlying this lifestyle.

**Methods:**

We integrated stable isotope (δ^13^C and δ^15^N) analyses, MIG-seq (multiplexed ISSR genotyping by sequencing)-based phylogenetics and fungal metabarcoding to investigate the physiological ecology and evolutionary history of three *Odontochilus* taxa: the large-leaved *O. fissus*, the small-leaved *O. nakaianus* (including albino, chlorophyll-deficient variants) and the very small-leaved *O.* aff. *fissus*. Morphologically, *O.* aff. *fissus* differs from *O. fissus* in having reduced, often reddish scale leaves and coralloid rhizomes, which are traits commonly observed in fully mycoheterotrophic orchids or mixotrophic orchids with high heterotrophy.

**Key Results:**

Albino individuals and protocorms of *O. nakaianus* exhibited isotope signatures characteristic of full mycoheterotrophy, whereas normal individuals of *O. fissus*, *O. nakaianus* and *O.* aff. *fissus* displayed isotopic patterns indicative of partial mycoheterotrophy, with fungal dependence probably inversely correlated with leaf size. Metabarcoding revealed that all taxa consistently associated with Ceratobasidiaceae operational taxonomic units, suggesting that similar rhizoctonia fungi support varying degrees of mycoheterotrophy. MIG-seq analysis confirmed that *O.* aff. *fissus*, *O. fissus* and *O. nakaianus* form distinct genetic clusters, while albino *O. nakaianus* individuals were genetically indistinguishable from their green counterparts.

**Conclusions:**

These findings provide evidence of both genetic and nutritional divergence between *O. fissus* and *O.* aff. *fissus*. The results expand our understanding of the mycoheterotrophic continuum in *Odontochilus* species associated with rhizoctonia fungi.

## INTRODUCTION

Mycoheterotrophy, in which plants acquire carbon and essential nutrients from mycorrhizal fungi, is common among orchids and other dust-seeded taxa (Merckx, 2013). Many orchids exhibit partial mycoheterotrophy or mixotrophy by combining photosynthesis with fungal carbon uptake (Gebauer and Meyer, 2003; Bidartondo *et al.*, 2004; Julou *et al.*, 2005). Stable isotope analyses of δ^13^C and δ^15^N (and more recently δ^2^H) are effective for evaluating this nutritional strategy, as mycoheterotrophic orchids generally show higher δ^13^C, δ^15^N, and δ^2^H values than co-occurring autotrophic plants (Gebauer and Meyer, 2003; Gebauer *et al.*, 2016). These isotopic trends indicate that partial mycoheterotrophy spans a continuum from minimal to near-complete fungal reliance, reflecting a stepwise evolutionary transition (Roy *et al.*, 2013; Suetsugu *et al.*, 2018; Selosse *et al.*, 2025).

Leaf traits such as size and pigmentation often correlate with fungal dependence (Leake, 1994; Tsukaya, 2018). Species with larger leaves typically show moderate ^13^C enrichment, while those with small, pale leaves tend to exhibit higher δ^13^C values, consistent with increased heterotrophy (Girlanda *et al.*, 2005; Zimmer *et al.*, 2008; Suetsugu *et al.*, 2018; Kobayashi *et al.*, 2021). For example, in *Cephalanthera*, species with well-developed foliage may obtain nearly 50 % of their carbon from fungi, whereas *C. subaphylla*, with very small leaves, depends predominantly on fungal sources (Sakamoto *et al.*, 2016). A similar trend occurs in *Pyrola* (Ericaceae), where the leafless *P. aphylla* is fully mycoheterotrophic or mixotrophic with high heterotrophy, whereas *P. picta*, which has well-developed leaves, is nearly autotrophic (Hynson *et al.*, 2009). Furthermore, partially mycoheterotrophic orchids with variegated leaves often display a negative correlation between chlorophyll content and carbon gain from fungi, with pale leaf sectors exhibiting greater ^13^C enrichment (Stöckel *et al.*, 2011; Suetsugu and Matsubayashi, 2025*b*).

Red or purple pigmentation is common in mixotrophic plants with high fungal reliance and may mitigate stress from reduced photosynthesis (Girlanda *et al.*, 2005; Hynson *et al.*, 2009; Suetsugu *et al.*, 2018, 2024). This pigmentation often coincides with low chlorophyll and high anthocyanin levels (Girlanda *et al.*, 2005; Suetsugu *et al.*, 2018). Although these traits are linked to reduced photosynthetic capacity (Girlanda *et al.*, 2005; Kobayashi *et al.*, 2021), anthocyanins may protect against light-induced oxidative damage (Sarma and Sharma, 1999). This pinkish coloration from anthocyanins is often seen in albino (chlorophyll-deficient) variants of partially mycoheterotrophic plants (Selosse *et al.*, 2004; Shutoh *et al.*, 2020).

High degrees of mycoheterotrophy are often correlated with specialized underground structures, such as coralloid rhizomes. The leafless genus *Corallorhiza* consistently produces branched coralloid rhizomes that enhance fungal contact and carbon uptake, while its leafy sister genus *Oreorchis* rarely develops such structures (MacDougal and Dufrenoy, 1944; Suetsugu and Okada, 2025*a*). Similarly, *Cremastra appendiculata*, a partially mycoheterotrophic species, sometimes develops such rhizomes in association with high fungal dependence (Yagame *et al.*, 2021, 2024; Zahn *et al.*, 2022; Suetsugu *et al.*, 2022). The morphological similarity to protocorms has led to the view that they function as persistent protocorms, retaining traits of the seedling stage optimized for fungal nutrition (Freudenstein, 1994; Suetsugu and Matsubayashi, 2021*a*; Suetsugu and Okada, 2025*a*).

The nutritional strategies of orchids are also influenced by their fungal partners (Selosse *et al.*, 2004; Julou *et al.*, 2005; Ogura-Tsujita *et al.*, 2012; Suetsugu *et al.*, 2022). Associations with ectomycorrhizal (ECM) or saprotrophic non-rhizoctonia fungi generally promote high levels of mycoheterotrophy, whereas most green orchids form symbioses with non-ECM rhizoctonias (hereafter rhizoctonias) (Wang *et al.*, 2021). Although δ^2^H studies suggest that partial mycoheterotrophy is common among rhizoctonia-associated orchids (Gebauer *et al.*, 2016; Fay *et al.*, 2018; Schiebold *et al.*, 2018; Yagi *et al.*, 2024), they usually acquire less fungal carbon than ECM-associated species (Schweiger *et al.*, 2018). In addition, fully mycoheterotrophic orchids relying solely on rhizoctonias are yet to be confirmed except in albino mutants. In some orchid groups, increased fungal dependence is accompanied by dual associations with ECM and rhizoctonia fungi, possibly facilitating transitions toward full mycoheterotrophy (Ogura-Tsujita *et al.*, 2012; Yagame *et al.*, 2021; Selosse *et al.*, 2022; but see Wang *et al.*, 2023).

To date, *Stigmatodactylus sikokianus* and *Disperis neilgherrensis* are the only known species to derive nearly 90 % of their carbon from rhizoctonias under natural conditions (Suetsugu *et al.*, 2021*a*, 2025*a*, *b*). While mutualistic mycorrhizal associations are often diffuse (Smith and Read, 2008), fully mycoheterotrophic plants often display high fungal specificity, probably favouring more beneficial partners (Bruns *et al.*, 2002; Egger and Hibbett, 2004; Hynson *et al.*, 2009). Both species associate with a narrow range of rhizoctonias, suggesting specialization toward lineages from which carbon is more easily extracted (Selosse *et al.*, 2025). However, fungal communities in related species with lower dependence remain poorly understood, making it unclear whether such specialization coevolves with mycoheterotrophy.

**Fig. 1.**
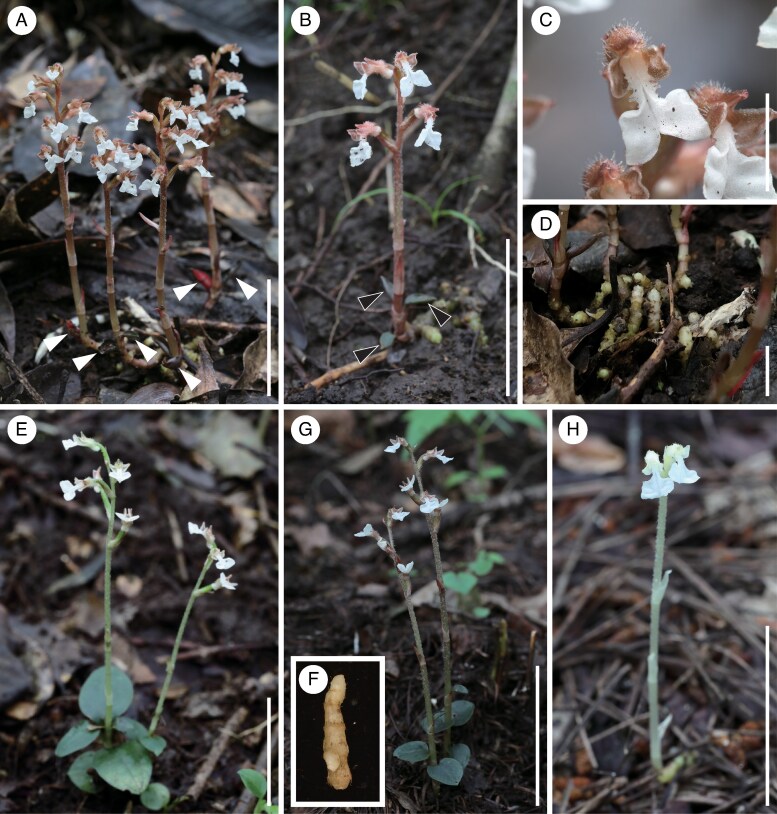
*Odontochilus* species investigated in this study. (A–B) Flowering plants of *Odontochilus* aff. *fissus*. (C) Close-up of *Odontochilus* aff. *fissus* flowers. (D) Coralloid rhizome of *Odontochilus* aff. *fissus*. (E) *Odontochilus fissus*. (F) Protocorm of *Odontochilus nakaianus.* (G) Green individuals of *Odontochilus nakaianus*. (H) Albino individual of *Odontochilus nakaianus*. Scale bars: 3 cm (A–B, E & G–H) and 5 mm (C–D).

In this context, the genus *Odontochilus* serves as an excellent model to investigate how degrees of mycoheterotrophy relate to morphological and physiological traits in green orchids probably associated with rhizoctonias. Species in this genus probably range from predominantly autotrophic (e.g. *O. putaoensis*) to fully mycoheterotrophic (e.g. *O. saprophyticus* and *O. poilanei*) (Tian *et al.*, 2014; Aung *et al.*, 2018). These orchids often occur in ECM-depleted understories, where rhizoctonia symbioses probably prevail (Suetsugu *et al.*, 2025*b*). This study focuses on *O. fissus* and *O.* aff. *fissus*, which coexist on Hachijo Island, Tokyo Prefecture, Japan. Typical *O. fissus* has green leaves longer than 1 cm and a slender rhizome (Maekawa, 1971; Satomi, 1982), whereas *O.* aff. *fissus* often bears scale-like red leaves (or small green leaves less than 5 mm) and a prominent coralloid rhizome, suggesting greater fungal reliance (Fig. 1). However, whether these forms represent distinct evolutionary lineages or phenotypic variants remains unclear, and no formal taxonomic treatment has been undertaken to date. Albino individuals of *O. nakaianus*, a sister taxon sometimes treated as conspecific with *O. fissus* (Ohwi and Kitagawa, 1983; Inoue, 2016), were also found in Saitama Prefecture. These albinos provide a reference for full mycoheterotrophy in *Odontochilus*, although such forms are often maladaptive in partially mycoheterotrophic species (Roy *et al.*, 2013; Lallemand *et al.*, 2019).

This study combines stable isotope (δ^13^C and δ^15^N) analyses, MIG-seq (multiplexed ISSR genotyping by sequencing)-based phylogenetics and fungal metabarcoding to explore the physiology and evolutionary history of *O.* aff. *fissus*, *O. fissus* and *O. nakaianus*. By comparing isotopic profiles with those of autotrophic plants and albino *O. nakaianus*, we estimated fungal carbon reliance. Fungal DNA from rhizomes was used to identify fungal partners, and MIG-seq was used to evaluate genetic differentiation and potential hybridization.

## MATERIALS AND METHODS

### Study species

*Odontochilus* aff. *fissus* and *O. fissus* are small terrestrial orchids that inhabit the shaded understories of Japanese forests. These species lack true roots and instead rely on rhizomes as the principal sites of mycorrhizal colonization. Taxonomic uncertainty surrounds *O. fissus* and *O. nakaianus*. Some authors limit the distribution of *O. nakaianus* to the Korean Peninsula, treating all Japanese individuals as *O. fissus* (Kitamura *et al.*, 1964; Nakajima, 2012). Others consider populations from both the Korean Peninsula and the Japanese islands of Honshu, Shikoku and Kyushu to belong to *O. nakaianus*, assigning the name *O. fissus* exclusively to specimens from the Izu Islands (Satomi, 1982; Yukawa, 2015). Still others have treated *O. fissus* and *O. nakaianus* as a single species (Ohwi and Kitagawa, 1983; Inoue, 2016).

In accordance with Satomi (1982) and Yukawa (2015), we identified individuals from the Izu Islands as *O. fissus*, differentiating them from *O. nakaianus* based primarily on geographical distribution and leaf length (9–13 mm in *O. fissus* vs. 3–7 mm in *O. nakaianus*). However, given the ambiguities mentioned above, we sampled *O. nakaianus* extensively throughout Japan to clarify the phylogenetic position of our focal samples (*O.* aff. *fissus* and *O. fissus* from Hachijo Island, and green and albino *O. nakaianus* from Kodama County, Saitama Prefecture; Fig. 1), using MIG-seq analysis (see below).

### Field study

In July 2022, we conducted a field survey on Hachijo Island, Tokyo Prefecture, where we observed ∼20 *O.* aff. *fissus* and 100 *O. fissus* individuals. Four 2 × 2-m quadrats, each containing at least one flowering *O.* aff. *fissus*, were established. To minimize the influence of microsite conditions, we also collected leaves from at least three autotrophic reference species of similar stature from surrounding 2 × 2-m quadrats (Gebauer and Meyer, 2003). In total, leaf samples were obtained from five *O.* aff. *fissus* (four bearing red, scale-like leaves and one with green leaves), three *O. fissus* and 12 reference plants. Additionally, one or two small rhizome segments (∼0.5 cm in length) were excised from each of five *O. fissus* individuals for subsequent molecular identification of mycorrhizal fungi.

A parallel survey was conducted in early August 2020 at an *O. nakaianus* population in Kodama County, Saitama Prefecture, which included roughly ten albino and 100 green individuals. Using similar sampling criteria, we collected three individuals per morph for mycorrhizal molecular analysis and seven individuals (four albino and three green), along with 12 autotrophic reference plants, for stable isotope analysis. In addition, putative protocorms located near adult *O. nakaianus* were sampled for both molecular and isotopic analyses (*n* = 2 for each).

Chlorophyll concentration and fluorescence (*F*_v_/*F*_m_) were measured in *O.* aff. *fissus*, *O. fissus*, green *O. nakaianus* and albino *O. nakaianus* (*n* = 4 per group) using a SPAD-502 chlorophyll meter (Konica Minolta Sensing Inc., Osaka, Japan) and a FluorPen FP100 fluorometer (Photon Systems Instruments, Brno, Czech Republic), following Shutoh *et al*. (2020) and Suetsugu *et al*. (2021*b*). Statistical differences were evaluated using Student’s *t*-tests.

### MIG-seq analysis

To resolve the phylogenetic identity of our focal taxa, we performed MIG-seq, a reduced-representation sequencing method. A sequencing library was constructed for 44 *Odontochilus* samples, including two *O.* aff. *fissus* from one population, eight *O. fissus* from two populations and 34 *O. nakaianus* (four of which were albinos) from eight populations (Supplementary Data Table S1), following the protocol of Suyama *et al.* (2022). Sequencing was conducted on an Illumina MiSeq using the MiSeq Reagent Kit v3 (75 bp × 2), and the resulting data were deposited in the DDBJ Sequence Read Archive (accession number PRJDB20701).

After adapter and primer trimming and quality filtering, 6 341 241 high-quality reads (mean ± SD: 144 119 ± 5579 per sample) were retained from 7 262 068 raw reads (165 047 ± 6630). *De novo* single nucleotide polymorphism (SNP) detection was performed using the Stacks 2.65 pipeline (Rochette *et al.*, 2019) with the following parameters: minimum stack depth (*m*) = 3, maximum stack distance (*M*) = 2, and catalogue mismatch allowance (*n*) = 2. SNPs with high heterozygosity (*H*_o_ ≥ 0.6) or fewer than three minor alleles were removed using the ‘populations’ module. Linkage disequilibrium was addressed using PLINK 1.90 (Chang *et al.*, 2015) with the option –indep-pairwise 50 100.1.

To assess genetic differentiation, we performed SNP-based maximum-likelihood phylogenetic and Neighbor-Net analyses. SNPs present in fewer than 50 % of individuals (*R* = 0.5) were excluded, yielding 1489 SNPs across 44 samples. Maximum-likelihood phylogenetic reconstruction was performed with RAxML 8.2.10 (Stamatakis, 2014) using the GTR substitution model, with Lewis’ ascertainment correction and 1000 bootstrap replicates. A Neighbor-Net network was also constructed based on an uncorrected p distance matrix excluding ambiguous sites, using SplitsTree4 4.14 (Huson and Bryant, 2006).

### Molecular identification of mycorrhizal fungi

Rhizome segments (∼0.5 cm) were hand-sectioned and microscopically examined to verify the presence of pelotons. Following surface sterilization, genomic DNA was extracted using a CTAB method (Doyle and Doyle, 1990).

Fungal ITS regions were amplified using two primer sets: ITS86F/ITS4, which broadly targets orchid mycorrhizal fungi, and ITS86F/ITS4Tul2, which specifically targets Tulasnellaceae fungi. Each primer included 3–6 random nucleotides and Illumina adapter sequences, following Suetsugu and Okada (2025*b*). A second PCR was conducted to add dual-indexed P5/P7 adapters and sample-specific barcodes (Syed *et al.*, 2009). Equimolar PCR products were pooled and sequenced using the MiSeq Reagent Kit v.2 (150 bp × 2). The raw sequences were submitted to the NCBI Sequence Read Archive under accession PRJNA1237408.

Bioinformatic processing was conducted using Claident v.0.9.2020.12.06 (Tanabe and Toju, 2013) as described by Suetsugu and Okada (2025*b*). Reads were trimmed, quality filtered, denoised using the integrated DADA2 pipeline, and screened for chimeras and index-hopping artefacts. High-quality reads were clustered into operational taxonomic units (OTUs) at 97 % similarity using VSEARCH 2.8.0, with the most abundant read representing each OTU. Taxonomic assignment was performed using the QCauto + LCA method against the Claident ‘overall_genus’ database. Singleton OTUs and sequences not associated with orchid mycorrhizae were excluded; only known orchid mycorrhizal partners (Dearnaley *et al.*, 2012; Wang *et al.*, 2021) were retained.

All *Odontochilus* specimens were dominated by OTUs affiliated with Ceratobasidiaceae. To contextualize these sequences, representative reads were aligned with related Ceratobasidiaceae accessions obtained via BLAST searches of the INSDC database using MAFFT v.7.475 (Katoh and Standley, 2013) with the L-INS-i option. A maximum-likelihood phylogeny was reconstructed using IQ-TREE v.2.2.2 (Nguyen *et al.*, 2015), with the best-fit substitution model selected via ModelFinder and node support assessed with 1000 SH-aLRT (Shimodaira–Hasegawa approximate likelihood ratio test) and ultrafast bootstrap replicates (Guindon *et al.*, 2010; Minh *et al.*, 2013).

### δ^13^C and δ^15^N analysis

Stable carbon and nitrogen isotope ratios in *Odontochilus* species and co-occurring autotrophic plants were measured using a continuous-flow isotope-ratio mass spectrometer (Delta V Advantage; Thermo Fisher Scientific, Waltham, MA, USA) coupled with an elemental analyser (Flash EA 2000; Thermo Fisher Scientific) following Suetsugu and Matsubayashi (2021*b*). Relative isotope abundances were calculated as:


$$\delta^{13}C\;\;\mathrm{or}\;\delta^{15}N=(R_{\mathrm{sample}}/R_{\mathrm{standard}}\text{–}1)\;\times 1000[\text{‰}],$$

where *R*_sample_ represents the ^13^C/^12^C or ^15^N/^14^N ratio of each sample, and *R*_standard_ corresponds to that of Vienna PeeDee Belemnite or atmospheric N_2_, respectively. Calibration was performed using laboratory standards CERKU 01, CERKU 02 and CERKU 03 (Tayasu *et al.*, 2011). Analytical standard deviations were <0.09 ‰ for ^13^C (*n* = 24) and <0.22 ‰ for ^15^N (*n* = 24). Total C and N concentrations were determined from sample weights and gas volumes of CO_2_ and N_2_ relative to the standards (Tayasu *et al.*, 2011). Enrichment factors (ɛ) were calculated as ɛ = δ_S_ − δ_REF_, where δ_S_ is the δ^13^C or δ^15^N value of an *Odontochilus* individual and δ_REF_ is the mean value for reference plants within the same plot (Preiss and Gebauer, 2008). The proportion of fungal-derived carbon (% Cdf) was estimated using a linear two-source mixing model: % Cdf = (ɛ^13^C_PMH_/ɛ^13^C_FMH_) × 100, where ɛ^13^C_PMH_ represents the ^13^C enrichment of a typical *Odontochilus* plant and ɛ^13^C_FMH_ is the mean enrichment of albino *O. nakaianus* plants.

A linear mixed model was used to compare δ^13^C and δ^15^N values among groups, with ‘plant identity’ (i.e. *O.* aff*. fissus*, *O. fissus*, albino and green *O. nakaianus*, *O. nakaianus* protocorms, and their reference plants) as a fixed factor and ‘plot’ as a random effect. *Post hoc* comparisons were performed using the Tukey–Kramer test. All statistical analyses were conducted in R (R Core Team, 2025) using the lme4 (Bates *et al.*, 2015) and multcomp (Hothorn *et al.*, 2008) packages.

## RESULTS

### Chlorophyll fluorescence and concentration

Chlorophyll content in *O.* aff. *fissus* (94.1 ± 30.4 mg m^−2^) was significantly lower than in *O. fissus* (330.1 ± 42.3 mg m^−2^; *P* < 0.001). However, both groups exhibited *F*_v_/*F*_m_ values within the typical range for autotrophic plants (0.7–0.83; Maxwell and Johnson, 2000; Ritchie, 2006), with no significant difference between *O.* aff. *fissus* (0.768 ± 0.017) and *O. fissus* (0.783 ± 0.022; *P* = 0.33).

In contrast, marked differences were found between green and albino *O. nakaianus*. Albino individuals had a mean chlorophyll concentration of 1.2 ± 0.1 mg m^−2^, while green individuals had 262.7 ± 22.1 mg m^−2^ (*P* < 0.001). *F*_v_/*F*_m_ values of green plants (0.785 ± 0.029) were within the autotrophic range, whereas albinos exhibited significantly reduced values (0.035 ± 0.029; *P* < 0.001), confirming their non-photosynthetic status.

### MIG-seq analysis

**Fig. 2.**
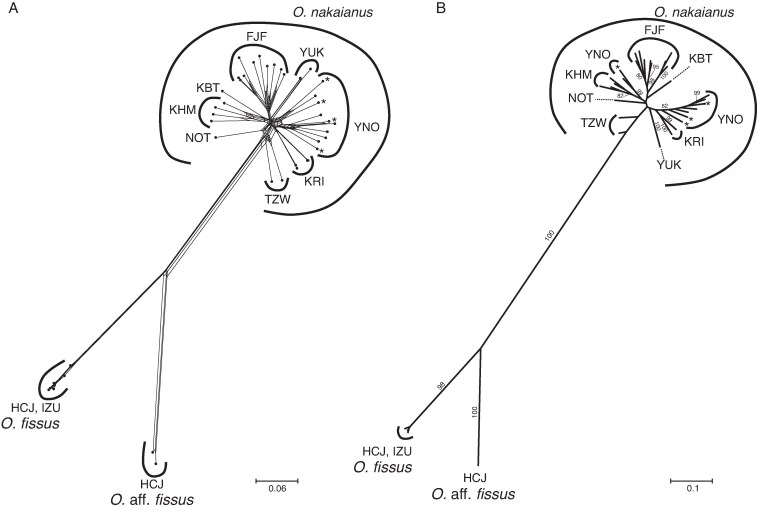
Phylogenetic tree of the *Odontochilus* species investigated, reconstructed from MIG-seq data. (A) Maximum-likelihood phylogeny. Nodes with bootstrap support values below 80 % are not shown. (B) Neighbor-Net network. Branch lengths represent the average number of substitutions per site. Albino *O. nakaianus* individuals are marked with an asterisk. Details of the sample IDs are provided in Supplementary Data Table S1.

MIG-seq-based maximum-likelihood phylogenetic analysis revealed that *O.* aff. *fissus* forms a distinct clade from sympatric *O. fissus* with 100 % bootstrap support. Similarly, Neighbor-Net analysis indicated that *O.* aff. *fissus* and *O. fissus* constitute distinct genetic clusters (Fig. 2).

Although some classifications have treated *O. nakaianus* and *O. fissus* as conspecific or assigned all Japanese specimens to *O. fissus* (Kitamura *et al.*, 1964; Nakajima, 2012; Inoue, 2016), our results support the genetic separation of *O. nakaianus* (from the mainland) and *O. fissus* (from the Izu Islands), affirming their status as distinct species (Fig. 2). No genetic differentiation was found between albino and green individuals of *O. nakaianus*, as the former clustered within the latter rather than forming a separate group.

### Molecular identification of mycobionts

**Fig. 3.**
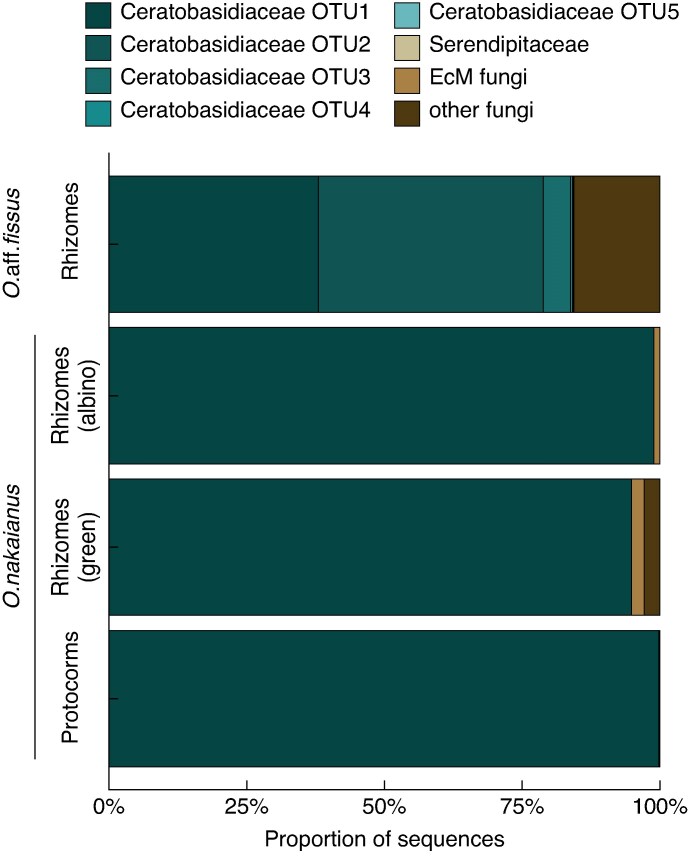
Relative abundance of mycorrhizal communities associated with *Odontochilus* species.

Metabarcoding revealed that all *Odontochilus* individuals consistently associated with Ceratobasidiaceae OTUs (Supplementary Data Table S2). The five *Odontochilus* aff. *fissus* specimens were dominated by three OTUs assigned to *Ceratobasidium* (OTU1, OTU2, OTU3), which together accounted for over 83 % of reads: OTU1 contributed 9589 reads (37.99 %), OTU2 10 302 reads (40.82 %) and OTU3 1255 reads (4.97 %). Likewise, OTU1 was overwhelmingly dominant in *O. nakaianus*, comprising 41 821 reads (98.87 %) in albino individuals, 22 945 (94.83 %) in green individuals and 26 000 (99.78 %) in protocorms (Fig. 3). One *O.* aff. *fissus* individual also yielded 3928 reads (15.56 %) assigned to the saprotrophic genus *Clitopilus*. ECM-associated OTUs were detected at low frequencies in *O. nakaianus*. *Russula* was represented by 401 reads (0.95 %) in albino plants, 124 (0.51 %) in green plants and 58 (0.22 %) in protocorms while *Sebacina* was detected in albino and green plants (75 reads, 0.18 %, and 428, 1.77 %, respectively) but was absent from protocorms.

**Fig. 4.**
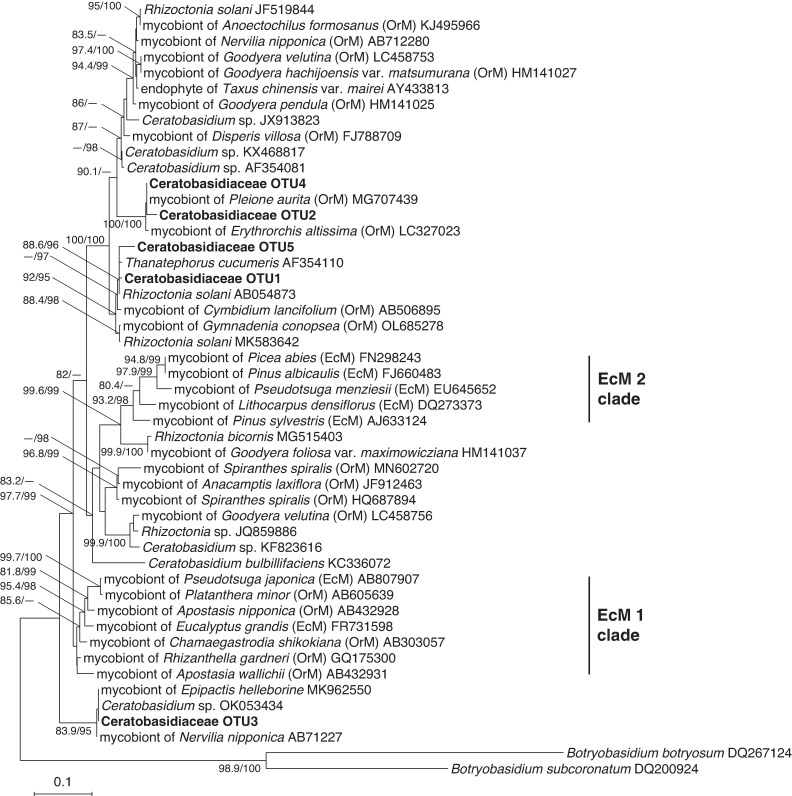
Phylogenetic tree of ITS2 rDNA sequences from Ceratobasidiaceae OTUs detected in mycorrhizal samples of *Odontochilus* species (in bold), together with sequences obtained from the INSDC database. The OTUs detected in *Odontochilus* species are ranked according to the number of sequencing reads. Accession numbers are provided for all INSDC sequences. The tree is rooted using *Botryobasidium botryosum* and *Botryobasidium subcoronatum* (Botryobasidiaceae). Nodes with SH-aLRT values below 80 % and ultrafast bootstrap values below 95 % are not shown. The scale bar indicates the number of substitutions per site. OrM: orchid mycorrhizal fungi; EcM: ectomycorrhizal fungi.

Phylogenetic analysis showed that these Ceratobasidiaceae OTUs formed supported monophyletic clades with known rhizoctonia-associated orchid mycobionts. Although ECM ability evolved independently in two Ceratobasidiaceae clades (EcM 1 and EcM 2 *sensu* Veldre *et al*., 2013), none of the OTUs identified here belonged to either group (Fig. 4). The dominant fungal partner probably corresponds to AG-2 BI, based on 98.7 % sequence identity with FJ492108, previously identified as AG-2 BI (Veldre *et al.*, 2013).

### δ^13^C and δ^15^N analysis

**Fig. 5.**
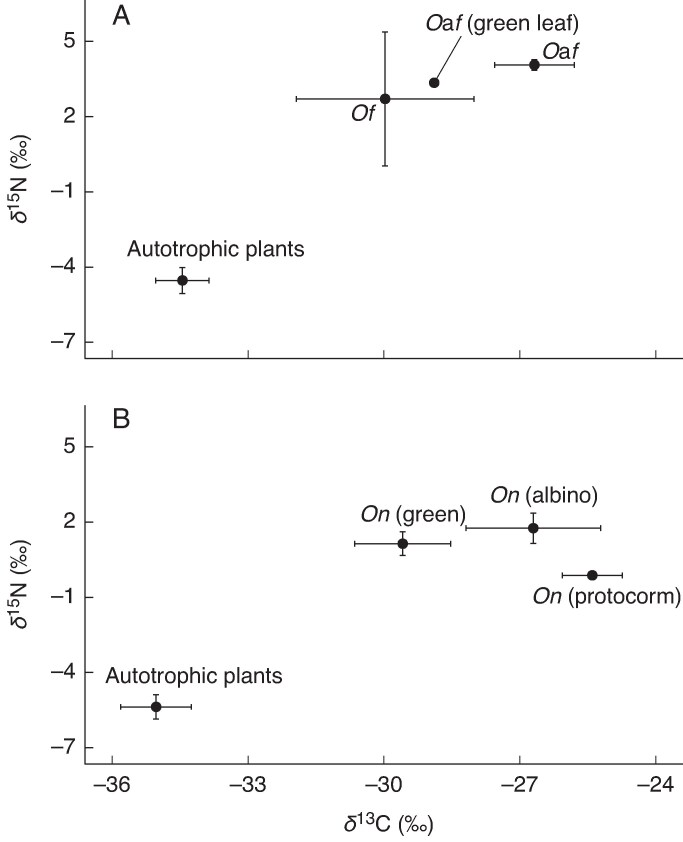
Mean (±SD) values of δ^13^C and δ^15^N in *Odontochilus* aff. *fissus* and *O. fissus* and their neighbouring autotrophic plants (A) and in albino and green *O. nakaianus* specimens and their neighbouring autotrophic plants (B). *O*a*f*: *Odontochilus* aff. *fissus*; *Of*: *Odontochilus fissus*; *On*: *Odontochilus nakaianus*.

The δ^13^C and δ^15^N values of *O.* aff. *fissus* (including both scale-leaved and green-leaved forms) and *O. fissus* were significantly higher than those of autotrophic reference plants (*P* < 0.001; Table 1). The δ^13^C values of scale-leaved *O.* aff. *fissus* were also significantly higher than those of *O. fissus* (*P* < 0.001), although no significant difference was observed in δ^15^N values between them (*P* = 0.31; Supplementary Data Table S3; Fig. 5).

**Table 1.** Mean (±SD) values of δ^13^C, δ^15^N and estimated fungal-derived carbon contributions for the *Odontochilus* species investigated here.

Taxon/phenotype	δ^13^C (‰)	δ^15^N (‰)	Fungal C (%)
Autotrophic reference plants	−34.4 ± 0.6	−4.5 ± 0.5	0
*O*. aff. *fissus* (scale-leaved)	−26.7 ± 0.9	4.1 ± 0.2	92.5 ± 10.6
*O.* aff. *fissus* (green-leaved)	−28.9	3.4	65.9
*O. fissus*	−30.0 ± 2.0	2.7 ± 2.7	53.3 ± 23.3
*O. nakaianus* (albino)	−26.7 ± 1.5	1.8 ± 0.6	100 (ref.)
*O. nakaianus* (green)	−29.6 ± 1.1	1.1 ± 0.5	64.9 ± 11.4
*O. nakaianus* protocorms	−25.4 ± 0.7	−0.1 ± 0.1	100

Albino *O. nakaianus* individuals showed significantly higher δ^13^C values than both green individuals and reference plants (*P* < 0.001; Supplementary Data Table S4), but did not differ significantly from protocorms (*P* = 0.41). All phenotypes of *O. nakaianus* (albino, green and protocorm) were significantly more enriched in ^15^N than autotrophic references (*P* < 0.001), with no significant difference between albino and green variants (*P* = 0.39; Fig. 5). Using the mean ^13^C enrichment of albino *O. nakaianus* as the baseline for full mycoheterotrophy, the estimated proportions of fungal-derived carbon were 64.9 ± 11.4 % in green *O. nakaianus*, 92.5 ± 10.6 % in scale-leaved *O.* aff. *fissus*, 65.9 % in green-leaved *O.* aff. *fissus* and 53.3 ± 23.3 % in *O. fissus*.

## DISCUSSION

Our study provides insights into the physiology, ecology and evolution of *O.* aff. *fissus*, *O. fissus* and *O. nakaianus* (including albino individuals) by integrating stable isotope (δ^13^C and δ^15^N) analyses, mycorrhizal fungal metabarcoding and MIG-seq-based phylogenetics.

Albino *O. nakaianus* exhibited pronounced ^13^C enrichment (8.4 ± 1.6 ‰), consistent with fully mycoheterotrophic protocorms of the same species and other fully mycoheterotrophic orchids (Zahn *et al.*, 2022). These isotopic signatures, combined with negligible chlorophyll fluorescence and very low chlorophyll concentrations, confirm that albino individuals rely primarily on fungal carbon. Protocorms of *O. nakaianus* showed even higher ^13^C enrichment (9.7 ± 1.1 ‰), possibly reflecting differences in carbon assimilation and metabolic fractionation between developmental stages. In addition, although albinism in partially mycoheterotrophic plants is often considered genetically fixed (Tranchida-Lombardo *et al.*, 2009), reversibility between albino and green morphs and the carryover of previously assimilated photosynthetic carbon may partially explain the slightly lower enrichment in albino individuals (Selosse *et al.*, 2025).

Albino morphs are typically regarded as maladaptive rather than transitional forms in the evolution of full mycoheterotrophy (Roy *et al.*, 2013; Lallemand *et al.*, 2019). Unlike evolutionary shifts that involve changes in mycorrhizal specificity toward fungi that support mycoheterotrophy (Jacquemyn and Merckx, 2019; Wang *et al.*, 2021), albino mutants in both ECM- and rhizoctonia-associated orchids usually retain the same fungal communities as their green counterparts (Selosse *et al.*, 2004; Julou *et al.*, 2005; Abadie *et al.*, 2006; Suetsugu *et al.*, 2017, 2019, 2021*b*; Suetsugu and Matsubayashi, 2025*b*). In line with this pattern, our metabarcoding results showed that green and albino *O. nakaianus* shared identical Ceratobasidiaceae OTUs. Moreover, phylogenetic analysis revealed no divergence between the two morphs, suggesting that albinism emerges sporadically within otherwise genetically cohesive populations.

In contrast, the transition toward near-complete mycoheterotrophy in *O.* aff. *fissus* probably occurred gradually, as reflected in morphological features such as reduced reddish scale leaves and coralloid rhizomes, indicative of a shift in resource allocation away from photosynthesis (Leake, 1994; Tsukaya, 2018). Notably, while *O.* aff. *fissus* has smaller leaves and lower chlorophyll content, its chlorophyll fluorescence remains similar to that of *O. fissus*. This contrasts with albino mutants where a sharp decline in chlorophyll content is accompanied by immediate loss of photosynthetic function (Julou *et al.*, 2005; Stöckel *et al.*, 2011). In many mixotrophic lineages, reductions in photosynthetic performance tend to follow, rather than precede, decreases in leaf size and chlorophyll content (Girlanda *et al.*, 2005; Cameron *et al.*, 2009; Suetsugu *et al.*, 2018, 2024). Our results for *O.* aff. *fissus* support this trajectory, consistent with an early stage of photosynthetic regression.

Variation in leaf size among *O. nakaianus*, *O. fissus* and *O.* aff. *fissus* corresponds closely with their degree of fungal carbon dependence. *Odontochilus fissus*, which has the largest leaves, obtains ∼50 % of its carbon from fungi, less than the smaller-leaved *O. nakaianus*. In contrast, *O.* aff. *fissus*, characterized by small, scale-like leaves and strong reddish pigmentation, exhibits the highest fungal dependence, with over 80 % of its carbon derived from fungi. Occasionally, *O.* aff. *fissus* individuals bear small but fully green leaves, and these plants show fungal dependence levels similar to green *O. nakaianus* (around 60–65 %). This pattern aligns with the hypothesis that progression toward obligate mycoheterotrophy is accompanied by reductions in leaf area to minimize carbon and water loss (Roy *et al.*, 2013), a trend observed in *Cephalanthera* and *Pyrola* (Hynson *et al.*, 2009; Sakamoto *et al.*, 2016; Shutoh *et al.*, 2016).

Phylogenetic analyses indicated that *O.* aff. *fissus* is genetically distinct from both *O. fissus* and *O. nakaianus*, despite overlapping distributions and flowering periods. A similar case has been documented in the *Pyrola picta* and *P. japonica* species complexes, where sympatric lineages with differing degrees of mycoheterotrophy are genetically and ecologically distinct and rarely hybridize (Hynson *et al.*, 2009; Jolles and Wolfe, 2012; Shutoh *et al.*, 2017), ultimately leading to taxonomic revision (Jolles and Wilson, 2014; Shutoh *et al.*, 2017). Although the observed genetic divergence could reflect sampling limitations, this seems unlikely given that *O. fissus* individuals from distant islands (Hachijo Island and Izu-Oshima Island, ∼180 km apart) formed a well-supported monophyletic clade. Nevertheless, broader geographical sampling is needed to clarify species boundaries and evaluate whether *O.* aff. *fissus* warrants formal taxonomic recognition.

Unlike many lineages that transition to full or near-full mycoheterotrophy, *O.* aff. *fissus* did not shift to ECM or saprotrophic non-rhizoctonia fungi. Although fungal specialization often accompanies the evolution of mycoheterotrophy (Jacquemyn and Merckx, 2019; Wang *et al.*, 2021; but see Roy *et al.*, 2009), *O.* aff. *fissus* retained associations with Ceratobasidiaceae OTUs, sharing its dominant mycorrhizal partner with *O. nakaianus*. Interestingly, *O.* aff. *fissus* appeared even less specialized than *O. nakaianus*, which consistently associated with a single OTU. The presence of a shared dominant fungus among *Odontochilus* species with differing levels of fungal dependence suggests that increased reliance on fungal carbon does not necessarily entail a shift in mycorrhizal partner identity.

Notably, *O.* aff. *fissus* exhibited pronounced ^13^C enrichment, a pattern rarely observed in orchids colonized by conventional rhizoctonia fungi. These findings imply that the associated fungi act as saprotrophic or ECM partners, at least under the environmental conditions at our study sites, facilitating net carbon transfer to the host. Nonetheless, given that green orchids associated with ECM-forming Ceratobasidiaceae exhibit markedly higher ^15^N enrichment (*Platanthera minor*: 11.4 ± 3.0 ‰; *Apostasia nipponica*: 21.6 ± 1.6 ‰) (Yagame *et al.*, 2012; Suetsugu and Matsubayashi, 2021*b*), the comparatively low ^15^N enrichment in the *Odontochilus* species examined (5.4–8.6 ‰) probably argues against ECM status and instead supports a saprotrophic identity for their fungal partners (Suetsugu *et al.*, 2025*b*).

Based on these findings, *O.* aff. *fissus* may be classified as a Type II mixotroph *sensu* Selosse *et al*. (2025), referring to species with substantial heterotrophic capacity that exploit rhizoctonia fungi possessing secondarily acquired saprotrophic or ECM traits. Both *O.* aff. *fissus* and albino *O. nakaianus* were predominantly associated with *Ceratobasidium* strains of AG-2 BI, a group known for trophic plasticity, including pathogenic potential (Carling *et al.*, 2002). While these fungi may represent undescribed obligate saprotrophic lineages, current evidence suggests that rhizoctonias associated with *Odontochilus* species primarily function saprotrophically under humid conditions, but are probably capable of occupying diverse ecological niches. To clarify their trophic role more precisely, future studies should include fungal isolation and inoculation experiments. Moreover, as net plant-to-fungus carbon transfer has been demonstrated in some rhizoctonia-associated orchids (Cameron *et al.*, 2006, 2008; Selosse *et al.*, 2025), transcriptomic analyses may help reveal the environmental and physiological factors governing carbon exchange directionality.

Overall, our integrated evidence indicates that a nutritional continuum exists within *Odontochilus*, with lineages differing in their degree of fungal reliance, and that *O.* aff. *fissus* represents a genetically distinct lineage with pronounced mycoheterotrophic tendencies. The persistence of rhizoctonia associations in *O.* aff. *fissus*, despite its high fungal dependence, highlights the potential of this genus as a model for exploring the evolution of mycoheterotrophy beyond ECM- or saprotrophic non-rhizoctonia lineages. As no fully mycoheterotrophic orchids are currently known to rely exclusively on non-ECM rhizoctonia fungi, further research is warranted to determine whether any *Odontochilus* species exhibit complete fungal dependence while maintaining these associations.

## Supplementary Material

mcaf100_Supplementary_Data

## Data Availability

MIG-seq data and fungal community data have been deposited in the Sequence Read Archive under accession numbers PRJDB20701 and PRJNA1237408, respectively. Additional supporting information is available online in the Supporting Information section at the end of this article.
